# Simultaneous Delivery of Curcumin and Resveratrol via In Situ Gelling, Raft-Forming, Gastroretentive Formulations

**DOI:** 10.3390/pharmaceutics16050641

**Published:** 2024-05-10

**Authors:** Worrawee Siripruekpong, Rachanida Praparatana, Ousanee Issarachot, Ruedeekorn Wiwattanapatapee

**Affiliations:** 1 Department of Pharmaceutical Technology, Faculty of Pharmaceutical Sciences, Prince of Songkla University, Hatyai, Songkhla 90112, Thailand; worraja@gmail.com; 2 Phytomedicine and Pharmaceutical Biotechnology Excellence Center, Faculty of Pharmaceutical Sciences, Prince of Songkla University, Hatyai, Songkhla 90112, Thailand; 3 Faculty of Medical Technology, Prince of Songkla University, Hatyai, Songkhla 90112, Thailand; rachanida.p@psu.ac.th; 4 Department of Pharmacy Technician, Faculty of Public Health and Allied Health Sciences, Sirindhorn College of Public Health Trang, Praboromarajchanok Institute, Kantang, Trang 92110, Thailand; ousaneemu@gmail.com

**Keywords:** in situ gelling, gastroretentive drug delivery system, Eudragit^®^ EPO, solid dispersion, curcumin, resveratrol

## Abstract

Curcumin and resveratrol are polyphenolic compounds that have been shown to exhibit synergistic therapeutic properties including anti-inflammatory, anticancer, and antiulcer activities, which may be exploited for the treatment of gastric diseases. However, both compounds have poor aqueous solubility and rapid metabolism, resulting in a low oral bioavailability. In situ gelling, liquid formulations were developed to produce a gastroretentive, raft-forming delivery vehicle to improve bioavailability. Solid dispersions containing a mixture of curcumin and resveratrol with Eudragit^®^ EPO (Cur/Res-SD) were first prepared using solvent evaporation, to improve the solubility and dissolution of the compounds. Solid dispersions of a weight ratio of 1:10 curcumin/resveratrol to Eudragit^®^ EPO were subsequently incorporated into in situ gelling, liquid formulations based on the gelling polymers, sodium alginate (low viscosity and medium viscosity), pectin, and gellan gum, respectively. Calcium carbonate and sodium bicarbonate were included to produce carbon dioxide bubbles in the gel matrix, on exposure to gastric fluid, and to achieve flotation. Moreover, the calcium ions acted as a crosslinking agent for the hydrogels. Optimized formulations floated rapidly (<60 s) in simulated gastric fluid (pH = 1.2) and remained buoyant, resulting in the gradual release of more than 80% of the curcumin and resveratrol content within 8 h. The optimized formulation based on medium-viscosity sodium alginate exhibited enhanced cytotoxic activity toward human gastric adenocarcinoma cell lines (AGS), compared with unformulated curcumin and resveratrol compounds, and increased anti-inflammatory activity against RAW 264.7 macrophage cells compared with the NSAID, indomethacin. These findings demonstrate that in situ gelling, liquid formulations, loaded with a combination of curcumin and resveratrol in the form of solid dispersions, show potential as gastroretentive delivery systems for local and systemic effects.

## 1. Introduction

Curcumin is a polyphenolic compound extracted from turmeric rhizomes (*Curcuma longa* Linn.) in the Zingiberaceae family [1]. It has been shown to possess pharmacological activities including gastroprotective, antioxidant [2], anti-inflammatory [2,3], anti-carcinogenic [4,5,6], anti-ulcerogenic [7], and anti-*Helicobacter pylori* [8,9] activities [10]. Several studies using gastric ulcer models [11] have shown that curcumin can decrease gastric acid secretion and inhibit ulceration by blocking the H_2_ histamine receptor [12]. Curcumin is considered to reduce inflammation through the inhibition of COX-2 expression and prostaglandin E2 synthesis (PGE-2) and by suppressing inducible nitric oxide synthase (iNOS)-mediated inflammation [13]. The intake overdose and long-term administration of curcumin can induce hepatotoxicity [14]. Therefore, the European Food Safety Authority (EFSA) has set curcumin’s Acceptable Daily Intake (ADI) at 3 mg/kg/day.

Resveratrol (3,5,4′-trihydroxy-trans-stilbene) is a natural polyphenolic compound extracted from grapes (*Vitis vinifera*), blueberries, cranberries, mulberries, and red wine. Numerous studies have shown that resveratrol exhibits a wide range of pharmacological activities including anti-inflammatory [15], antioxidant [16], antiulcer [17], and anti-carcinogenic behavior [18]. Resveratrol has been reported to exert anti-gastric and colorectal cancer activities, through anti-proliferative action and the induction of apoptotic responses [19]. Murine models of *Helicobacter pylori* (*H. pylori*) infection showed that resveratrol inhibited gastric inflammation [20] through the suppression of interleukin-8 (IL-8), inducible nitric oxide synthase (iNOS), and nuclear factor-kappa B (NF-κB). The compound has also been reported to show protective and therapeutic effects on acetic acid-induced [21] and indomethacin-induced gastric ulcers in rats [22].

The combination of curcumin and resveratrol has shown synergistic efficacy for treating gastrointestinal diseases like gastric cancer and colorectal cancer. Curcumin and resveratrol possess strong antioxidant properties that protect cells against oxidative damage [4]. The antioxidant effects of curcumin with resveratrol were enhanced four times more than those of curcumin with quercetin. The combination of curcumin and resveratrol was found to be more effective than individual compounds in activating the p53 tumor suppressor against gastric cancer [23]. In addition, curcumin and resveratrol combinations have been shown to give rise to pronounced synergistic effects [24], resulting in the inhibition of colon cancer cell growth via the deactivation of EGFR and the stimulation of cell apoptosis [25]. Accordingly, the combination of curcumin and resveratrol exhibits much promise for the treatment of gastrointestinal diseases.

However, both compounds have found limited clinical utility to date, due to poor bioavailability and stability in acidic environments. Curcumin has an extremely low aqueous solubility (0.6 µg/mL) and a short gastric residence time [26,27]. It is incompletely absorbed and has a poor oral bioavailability of about 60%, due to hepatic first-pass metabolism. Resveratrol exhibits poor aqueous solubility (40 µg/mL) [28] and poor oral bioavailability (<1%), accentuated by its short half-life and labile character. This compound is rapidly metabolized and eliminated, due to hepatic glucuronidation and sulfation.

Several formulation approaches have been investigated to enhance the solubility of curcumin and resveratrol including nanoparticle formation, self-microemulsification molecular complexation, and solid dispersion [29], which incorporates the active substance within an inert hydrophilic carrier.

Gastroretentive drug delivery devices often involve the in situ formation of a raft structure following the oral administration of a gel-forming, liquid formulation, which floats on the stomach content and prolongs drug release, thereby resulting in enhanced absorption or local action. The formulation typically consists of a natural gel-forming polymer and a gas-generating agent, calcium carbonate (CaCO_3_), and sodium bicarbonate (NaHCO_3_). Following contact of the gel-forming liquid with gastric fluid, the formation and entrapment of gas bubbles within the gel raft structure lowers the density and maintains buoyancy. The raft layer functions as a physical barrier that helps alleviate esophagitis, heartburn, and gastroesophageal reflux disease (GERD) by preventing the backflow of stomach contents into the esophagus [30]. Moreover, the characteristics of in situ gelling formulations are relatively suitable for children, the elderly, and patients with dysphagia problems. It can act as a pH-neutral barrier to provide a significant acid-neutralization capacity, similar to antacids. Nevertheless, different gastroretentive systems such as bioadhesive systems, expandable systems, and high-density systems do not provide this physical barrier characteristic.

The natural gel-forming biopolymers pectin, alginate, and gellan gum, having advantages of biodegradability and biocompatibility, are prime candidates for raft-forming formulations. Pectin is an anionic polysaccharide consisting of α-(1–4)-linked D-galacturonic acid and 1, 2-linked L-rhamnose that is extracted from the cell wall of plants such as citrus fruits. Sodium alginate is a linear block copolymer polysaccharide, extracted from seaweed and consisting of β-d-mannuronic acid and α-l-glucuronic acid, linked by 1,4-glycosidic bonds. Sodium alginate and pectin form hydrogels through the crosslinking of polymer chains using divalent or trivalent cations. Gellan gum is a microbial exopolysaccharide produced by *Shingomonas paucimobilis* and *Sphingomonas elodea*. Gelation occurs by complexation with divalent cations in a double helical structure [31].

Raft-forming formulations have been reported to successfully deliver various drugs and herbal medicines. For example, etoricoxib–famotidine was combined and was beneficial for the treatment of pain and preventing ulcers in long-term use [32]. Raft-forming formulations of several herbal medicines also supported the effectiveness of this system such as curcumin [33,34] chebulinic acid [35], *Aloe vera* [36], and quercetin [37]. Additionally, some of them, raft-forming of *Centella asiatica* extract [38] and curcumin [33], reached in vivo tests. Among these formulations, the raft-forming system was different in terms of the types and the amount of gelling agent and retard polymer. Most previous research has focused on single herbal medicine-loaded raft-forming formulations. Therefore, in this study, it was interesting to find out the suitable raft-forming system for the simultaneous delivery of two synergistic bioactive compounds, curcumin and resveratrol.

The aim of the research was to enhance the dissolution of curcumin and resveratrol in the stomach by developing an in situ gelling, gastroretentive, raft-forming formulation incorporating solid dispersions containing a combination of curcumin and resveratrol. Eudragit^®^ EPO is a synthetic cationic copolymer composed of dimethyl aminoethyl methacrylate, butyl methacrylate, and methyl methacrylate (ratio = 2:1:1) and was utilized as the hydrophilic carrier in the solid dispersion. Eudragit^®^ EPO is commonly used as a solubility-enabling excipient [39] for anionic drugs at pH < 5. Sodium alginate (low- and medium-viscosity types), pectin, and gellan gum were investigated as the gel-forming polymer. The physicochemical characteristics of the in situ gelling, raft-forming formulations and the formed raft structures were investigated in depth and their biological performance (anti-inflammatory effect and cytotoxic activity) was evaluated in cell culture.

## 2. Materials and Methods

### 2.1. Materials

Curcuminoid extract powder derived from *Curcumin longa* (96.1% curcumin) was purchased from Thai-China Flavours and Fragrances Industry Co., Ltd. (Phra Nakhon Si Ayutthaya, Thailand). Resveratrol (3,5,4′-trihydroxy-trans-stilbene; purity > 98%) was purchased from Pioneer Herb (Shanghai, China). Eudragit^®^ EPO was provided by Evonik Industries AG (Essen, Germany). Sodium alginate (medium viscosity) was obtained from High Science Limited Partnership (Songkhla, Thailand). Sodium alginate (low viscosity) and gellan gum (Gelzan™ CM) were purchased from Sigma-Aldrich, Inc. (St. Louis, MO, USA). Pectin (GENU^®^), low methoxy grade, was sourced from CP Kelco, Inc. (Atlanta, GA, USA). Sodium bicarbonate and ethanol were provided by RCI Labscan (Bangkok, Thailand). Calcium carbonate was obtained from Vidhayasom Co., Ltd. (Bangkok, Thailand). Polyvinylpyrrolidone (PVP K30) was purchased from P.C. Drug Center Co., Ltd. (Bangkok, Thailand). All reagents were of pharmaceutical or analytical grade.

### 2.2. Cell Culture

The human gastric adenocarcinoma cell line (AGS; CRL:1739™) and murine macrophage cell line (RAW264.7, TIB-71™) were supplied by the American-Type Culture Collection (ATCC^®^, Manassas, VA, USA). Penicillin-streptomycin, fetal bovine serum (FBS; 10%), trypsin EDTA (0.25%), and Kaighn’s Modification of Ham’s F-12 Medium (F-12K) were supplied by Gibco™ (Invitrogen, CA, USA). MTT (3-(4,5-dimethylthiazol-2-yl)-2,5-diphenyltetrazolium bromide) is a colorimetric reagent sourced from Invitrogen™ (Thermo Fisher Scientific, Eugene, OR, USA) for the analysis of viability and cytotoxicity assays.

Roswell Park Memorial Institute (RPMI-1640) medium, 10% FBS, penicillin (100 IU/mL), streptomycin (100 mg/mL) solution, phosphate-buffered saline (PBS; pH = 7.4), and trypsin-EDTA 0.25% were obtained from Gibco™ (Invitrogen, CA, USA). Lipopolysaccharide (LPS), extracted from Escherichia coli, Indomethacin, and modified Griess reagent, was purchased from Sigma-Aldrich (Saint Louis, MO, USA). Dimethyl sulfoxide (DMSO) was provided by RCI Labscan (Bangkok, Thailand). All other reagents were of analytical or pharmaceutical grade.

### 2.3. Methods

#### 2.3.1. Preparation of Solid Dispersions Containing a Mixture of Curcumin and Resveratrol

Solid dispersions containing a mixture of curcumin (Cur) and resveratrol (Res) were prepared using the solvent evaporation method. Curcumin and resveratrol were combined at a *w*/*w* ratio of 1:1 and were dissolved in ethanol, resulting in a clear solution. Eudragit^®^ EPO was added to the co-solution to obtain Cur/Res: EPO at *w*/*w* ratios of 1:0.5, 1:1, 1:3, 1:5, 1:6, 1:8, and 1:10 (Table 1). The solvent was removed using a rotary evaporator (Hei-VAP Value, Heidolph Instruments, Schwabach, Germany) at 40 °C for 4–8 h. The dried Cur/Res solid dispersion (Cur/Res-SD) was removed from the reservoir, pulverized using a pestle and mortar, and passed through a 250 µm mesh to obtain a particle size range of 0.05–0.25 mm.

Curcumin/resveratrol physical mixtures (Cur/Res-PM) were prepared at the same *w*/*w* ratio via thorough mixing and reduction using a mortar and pestle before passing through 250 µm mesh to obtain a fine powder. Cur/Res-SDs and Cur/Res-PMs were stored in tight, light-resistant containers at room temperature in a vacuum desiccator until used for analysis.

#### 2.3.2. Physicochemical Characteristics of Solid Dispersion Containing a Mixture of Curcumin and Resveratrol

##### Dissolution Behavior

The dissolution behavior of curcumin, resveratrol, Cur/Res-SDs, and Cur/Res-PMs was investigated according to the USP 41/NF36 monograph [33]. Each test sample was accurately weighed to ensure that it contained 10 mg of curcumin or resveratrol [33]. The dissolution tester (EDT 08Lx, Electrolab, Mumbai, India) was equipped with a rotating paddle (apparatus II, 50 rpm rotation speed) and testing was carried out at 37 ± 0.5 °C. Simulated gastric fluid (0.1 N hydrochloric acid, pH = 1.2, 900 mL) was used as the dissolution medium. Samples (5 mL) were withdrawn and replaced with fresh medium at time intervals of 5, 10, 15, 30, 45, 60, 90, and 120 min and the content of curcumin and resveratrol was assayed using UV–visible spectrophotometry (UV-1800, Bara Scientific Co., Ltd., Bangkok, Thailand) at wavelengths of 306 nm and 425 nm, respectively. Each sample was tested in triplicate (*n* = 3) and data were reported as the mean value ± S.D. Dissolution profiles were generated by plotting the percentage cumulative dissolution (%*w*/*w*) against time.

##### Powder X-ray Diffraction (PXRD)

Powder X-ray diffraction studies were carried out to confirm the crystalline or amorphous structure of curcumin and resveratrol in unformulated compounds, solid dispersions, and physical mixtures, respectively. All samples were measured (Powder X-ray diffractometer [PXRD]; Philips: X’ pert MPD, Amsterdam, The Netherlands) at room temperature. Diffraction patterns were generated under a voltage of 40 kV and a current of 30 mA. Each sample was analyzed using a step size of 0.05° over a scan range (*2θ*) of 5–90°, at a scan speed of 1 s/step for 2 h.

##### Fourier Transform Infrared Spectroscopy (FTIR)

FTIR studies were performed to identify functional groups and chemical interactions involving curcumin, resveratrol, and the Eudragit^®^ EPO hydrophilic carrier polymer. Spectra for curcumin, resveratrol, Cur/Res-SDs, and Cur/Res-PMs were generated using a spectrometer (VERTEX 70, Bruker, Ettlingen, Germany) equipped with a deuterated triglycine sulfate detector. Each sample was mixed with potassium bromide (KBr) and compressed into a disk using hydrostatic pressure, prior to scanning over the range of 400 to 4000 cm^−1^ with a resolution of 4 cm^−1^.

#### 2.3.3. Preparation of In Situ Gelling, Raft-Forming, Liquid Formulations Containing a 1:1 Mixture of Curcumin and Resveratrol

In situ gelling, raft-forming formulations were prepared using Cur/Res-SD and different types and concentrations of gelling polymer. Sodium bicarbonate (NaHCO_3_) and calcium carbonate (CaCO^3^) were added as CO_2_-generating agents. Briefly, the gelling polymer was dissolved in 100 mL of deionized water containing sodium bicarbonate (0.50% *w*/*v*), with stirring, to obtain a clear solution. Calcium carbonate (0.75% *w*/*v*) was added and mixing was continued, resulting in a homogenous solution. Finally, 2.5 g of Cur/Res-SDs (1:10 *w*/*w* ratio Cur/Res:EPO) was incorporated into the viscous solution and was thoroughly mixed for 4–8 h. The resulting formulations were stored in tight, light-resistant containers until further use. In the case of pectin and gellan gum formulations [40], the polymer solution containing sodium bicarbonate (100 mL) was heated to 40–50 °C and 90 °C, respectively, for complete dissolving, before adding calcium carbonate and active ingredients. The polymer solutions were cooled below 40 °C prior to the addition of Cur/Res-SD. The compositions of the various in situ gelling, raft-forming formulations are listed in Table 2.

#### 2.3.4. Physicochemical Properties of In Situ Gelling, Raft-Forming Formulations Containing a 1:1 Mixture of Curcumin and Resveratrol

##### Physical Appearance and pH Measurement

The physical appearance of in situ gelling, liquid formulations loaded with Cur/Res-SD was evaluated in terms of color and homogeneity [38]. The pH was measured using a digital pH meter at 25 ± 1 °C (FiveEasy™ F20 pH/mV, Mettler-Toledo GmBH, Greifensee, Switzerland).

##### Viscosity

The viscosity of in situ gelling, liquid formulations (20 mL sample volume) was measured at 25 ± 1 °C using a Brookfield digital viscometer (Model DV-III ULTRA, Amatek, MA, USA) fitted with spindle no. 64. Test samples in triplicate were subjected to spindle rotation over the range of 50–250 rpm for 30 s [33].

##### Floating Behavior

In situ gelling, liquid formulations (10 mL) were added to 100 mL of simulated gastric fluid (SGF, 0.1 N hydrochloric acid solution, pH = 1.2) at 37 °C. The floating lag time was recorded as the time taken for the formed gel to float to the surface of the medium. The floating duration time was the time that the gel remained afloat. Each liquid formulation was tested in triplicate [37].

##### Raft Density

The density of the raft formed by the gelling of the liquid formulation was determined using a measuring cylinder. Simulated gastric fluid (SGF; 35 mL) was added to a measuring cylinder and the weight was recorded (W_before_). In situ gelling, liquid formulation (10 mL) was added to the cylinder and allowed to form a gel on the SGF surface. The total weight of the gel raft and cylinder was measured after 30 min (W_after_). The volume of the gel raft was recorded from the measuring cylinder scale (V_r_) and the raft density was calculated using the following equation:

$$\mathrm{Raft}\;\mathrm{density}\;(g/\mathrm{mL})=\frac{\left(W_{\mathrm{after}}-{\;W}_{\mathrm{before}}\right)}{V_{r}}$$

Each formulation was tested in triplicate.

##### Raft Strength

Raft strength was measured using a texture analyzer (TA.XT plus, Stable Micro Systems; Charpa Techcenter Co., Ltd., Bangkok, Thailand) equipped with a 5 kg load cell. An L-shaped stainless-steel hook or probe (20 × 90 mm) was set in a vertical and centered position in a 250 mL glass beaker containing SGF (150 mL) [38]. The probe was attached to the texture analyzer and suspended in the lower third of the SGF medium, without contacting the beaker base. In situ gelling, liquid formulation (20 mL) was transferred into the beaker and was allowed to gel around the probe for 30 min. The L-shaped probe was subsequently drawn vertically through the gel raft at a speed of 5 mm/s and the maximum force (g) required to break through the raft was recorded as the raft strength.

##### Release of Curcumin and Resveratrol from In Situ Gelling, Liquid Formulations

Release of curcumin and resveratrol from the in situ gelling, liquid formulations was investigated using the dissolution apparatus II with paddle rotation set at 50 rpm (EDT 08Lx, Electrolab, Mumbai, India), according to the USP 41/NF36 monograph. The simulated gastric fluid release medium (900 mL) was maintained at 37 ± 0.5 °C. In situ gelling, liquid formulation (20 mL) was poured into the medium and 5 mL samples were withdrawn and replaced with the same volume of fresh medium at time = 30, 60, 120, 180, 240, 300, 360, 420, and 480 min. The amount of curcumin and resveratrol released, respectively, was measured using UV–visible spectrophotometry (UV-1800, Bara Scientific Co., Ltd., Bangkok, Thailand), as described above. Each formulation was tested in triplicate. Data were reported as mean ± SD and release profiles were constructed for each compound, as % cumulative release versus time.

The release kinetics were derived by fitting the release data to the zero-order, first-order, Higuchi, and Kormeyer–Peppas kinetic models [41]. $C_{0}$ is the amount of curcumin or resveratrol released at the initial time. *C_t_* is the amount released at time (t), while k is the release constant. A correlation coefficient (r^2^) close to 1 signifies the best fit of the actual release data to a particular kinetic model.

$$\begin{array}{cc} \mathrm{Zero order}: & C_{t}=C_{0}+\mathrm{kt}\\ \mathrm{First order}: & \ln C_{t}=\ln C_{0}+\mathrm{kt}\\ & \log C_{t}=\log C_{0}-(\mathrm{kt}/2.303)\\ \mathrm{Higuchi}: & C_{t}=k\sqrt{t}\\ & C_{t}={kt}^{½}\\ \mathrm{Kormeyer}\;–\;\mathrm{Peppas}: & \left[\frac{M_{t}}{M_{\infty }}\right]=kt^{n} \end{array}$$

where the n value characterizes the release mechanism (n ≤ 0.45 = Fickian diffusion mechanism, 0.45 < n ≤ 0.89 = non-Fickian transport, and n > 0.89 = super case II transport).

#### 2.3.5. Assay of Cytotoxic Activity Using Gastric Cancer Cell Lines

The cytotoxic activity of in situ gelling, liquid formulations containing both curcumin and resveratrol was measured using the MTT assay using AGS cells (human gastric adenocarcinoma cell line; ATCC^®^ CRL-1739™) [42]. MTT (3-(4,5-dimethylthiazol-2-yl)-2–5-diphenyltetrazolium bromide) is modified by mitochondrial reductase enzymes in viable cells to formazan (purple coloration). AGS cells were seeded at a cell density of 1 × 10^4^ cells/well in 96-well plates in F-12K medium supplemented with 10% FBS, 100 µg/mL streptomycin, and 100 U/mL penicillin. The cells were incubated at 37 °C in a humidified, 5% carbon dioxide (CO_2_) atmosphere for 24 h, before removing the medium and replacing with the test samples—curcumin; resveratrol; Cur/Res mixture; Cur/Res-SD (1:10); in situ gelling, liquid formulation based on medium-viscosity alginate and loaded with Cur/Res-SD (AM1); and in situ gelling, liquid formulation based on medium- viscosity alginate without active compounds (AM1 Blank). Test samples were prepared at various concentrations of curcumin and resveratrol (1.56–200 µg/mL) via dilution with culture medium and were applied to the AGS cells. Cell culture medium with fetal bovine serum served as the control group. After 24 h exposure, MTT solution (20 µL, 5 mg/mL in PBS) was added to each well and was incubated for a further 4 h. The supernatant was carefully removed and 200 μL of dimethyl sulfoxide (DMSO) was added to each well to solubilize the purple formazan crystals. The absorbance of formazan was measured at a wavelength of 570 nm, using a microplate reader (Biotek model PowerWave X, Santa Clara, CA, USA), and the percentage cell viability was calculated according to the following equation:

$$\mathrm{Cell}\;\mathrm{viability}\;(\%)=\left[\frac{{\mathrm{OD}}_{\mathrm{sample}}}{{\mathrm{OD}}_{\mathrm{control}}}\right]\times 100$$

where OD_sample_ and OD_control_ are the UV–visible absorbance readings at 570 nm wavelength of the test sample and control, respectively. IC_50_ values were assigned corresponding to the sample concentration that inhibited cell proliferation by 50%. Each formulation was tested in triplicate.

#### 2.3.6. Assay of Anti-Inflammatory Activity Using RAW264.7 Macrophage Cell Lines

The anti-inflammatory activity of in situ gelling, liquid formulations containing Cur/Res-SD was assessed via the indirect measurement of nitric oxide (NO) produced by murine macrophage cell lines (RAW264.7) [43,44]. Cells were cultured on 96-well plates at 1 × 10^5^ cells per well in RPMI medium, maintained at 37 °C in a 5% CO_2_ atmosphere. RPMI medium contained 100 units/mL penicillin G, 100 µg/mL streptomycin, and %10 FBS. After seeding for 24 h, the culture medium was removed and replaced with test sample either with or without 100 µg/mL of lipopolysaccharide (LPS). Test samples, including curcumin; resveratrol; Cur/Res-SD (1:10); in situ gelling, liquid formulation based on medium-viscosity alginate and loaded with Cur/Res-SD (AM1); and in situ gelling, liquid formulation based on medium-viscosity alginate without active compounds (AM1 Blank), were prepared at different concentrations in the range of 3.13 to 200 µg/mL, via dilution with culture medium. After incubation for 24 h, the supernatant of each well was collected and transferred into 96-well plates for the indirect measurement of nitric oxide. Griess reagent was added to the supernatant, to react with nitrite and produce azo compounds, which were detected at an absorbance wavelength of 570 nm [44]. Indomethacin was used as a positive anti-inflammatory control. The anti-inflammatory activity was expressed in terms of nitric oxide inhibition and was calculated using the following equation:

$$\mathrm{Inhibition}\;(\%)=\left[1-\left(\frac{D-B}{A-C}\right)\right]\times 100$$

in which the absorbance is detected at a wavelength of 570 nm when A is LPS (+), sample (−); B is LPS (+), sample (+); C is LPS (−), sample (−); D is LPS (−), sample (+); and A – C is NO_2_ concentration (µM)

#### 2.3.7. Statistical Analysis

The data were reported as average ± standard deviation and were analyzed using a one-way analysis of variation (ANOVA), using the SPSS program to identify differences between mean values. Statistical significance was denoted by *p*-values less than 0.05 (*p* < 0.05).

## 3. Results and Discussions

### 3.1. Physicochemical Characteristics of Curcumin and Resveratrol Solid Dispersions

#### 3.1.1. Dissolution Behavior of Curcumin and Resveratrol Solid Dispersions

**Figure 1:**
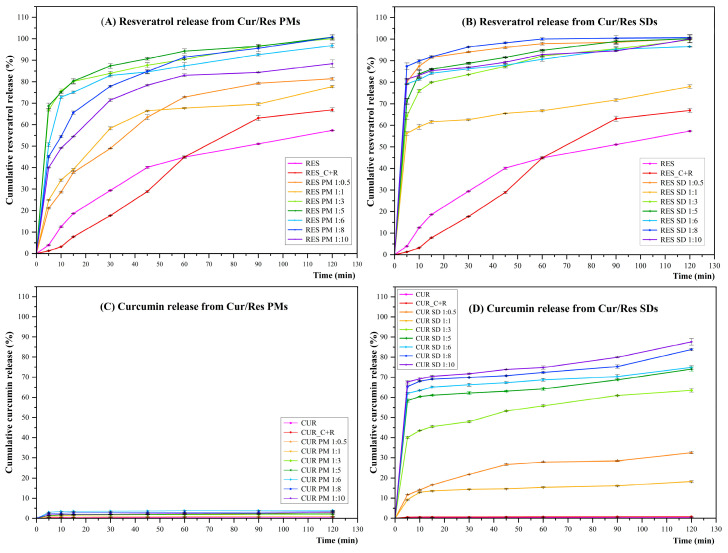
Dissolution profiles of curcumin and resveratrol from SDs and PMs in simulated gastric fluids at 37 °C.

Curcumin and resveratrol are polyphenolic compounds that exhibit poor aqueous solubility. To improve this property, curcumin and resveratrol were combined in solid dispersions using the hydrophilic carrier polymer, Eudragit^®^ EPO. The resulting SDs and PMs were obtained as orange-red powders with particle sizes in the range of 50–250 µm. The dissolution of curcumin (MW = 368.38 g/mol) in SGF was less than 1% within 2 h, due to the compound’s poor aqueous solubility (Figure 1C), hydrophobic nature, and low wetting properties. Curcumin dissolution from Cur/Res-PMs was slightly increased, but was still less than 4%. In contrast, the dissolution of curcumin from Cur/Res-SDs was dramatically increased and reached around 85% at 2 h for SDs prepared using a 1:10 *w*/*w* ratio of Cur/Res:EPO (Figure 1D). Around 57% dissolution of ‘non-formulated’ resveratrol (molecular weight = 228.25 g/mol) into SGF was measured after 2 h (Figure 1A). Dissolution increased when formulated with curcumin and Eudragit^®^ EPO as physical mixtures and attained a level of 100% for PMs prepared using Cur/Res:Eudragit^®^ EPO ratios of 1:3 and above. The dissolution of resveratrol from Cur/Res-SDs was more rapid than from PMs and was complete for Cur/Res: Eudragit^®^ EPO ratios above 1:3 (Figure 1A,B). The enhancement of curcumin and resveratrol solubility when formulated as solid dispersions with Eudragit^®^ EPO is considered to result from various mechanisms, including the inhibition of aggregation, reduction in crystal size, and improved wettability. Additionally, Eudragit^®^ EPO is a positively charged polymer that interacts with negatively charged curcumin for an improvement in water solubility. The maximum curcumin and resveratrol dissolution in SGF was obtained from SDs featuring a Cur/Res: Eudragit^®^ EPO *w*/*w* ratio of 1:10 and this design was used for the preparation of in situ gelling and raft-forming liquid formulations.

#### 3.1.2. Powder X-ray Diffraction Studies of Curcumin and Resveratrol Physical Mixtures and Solid Dispersions

**Figure 2:**
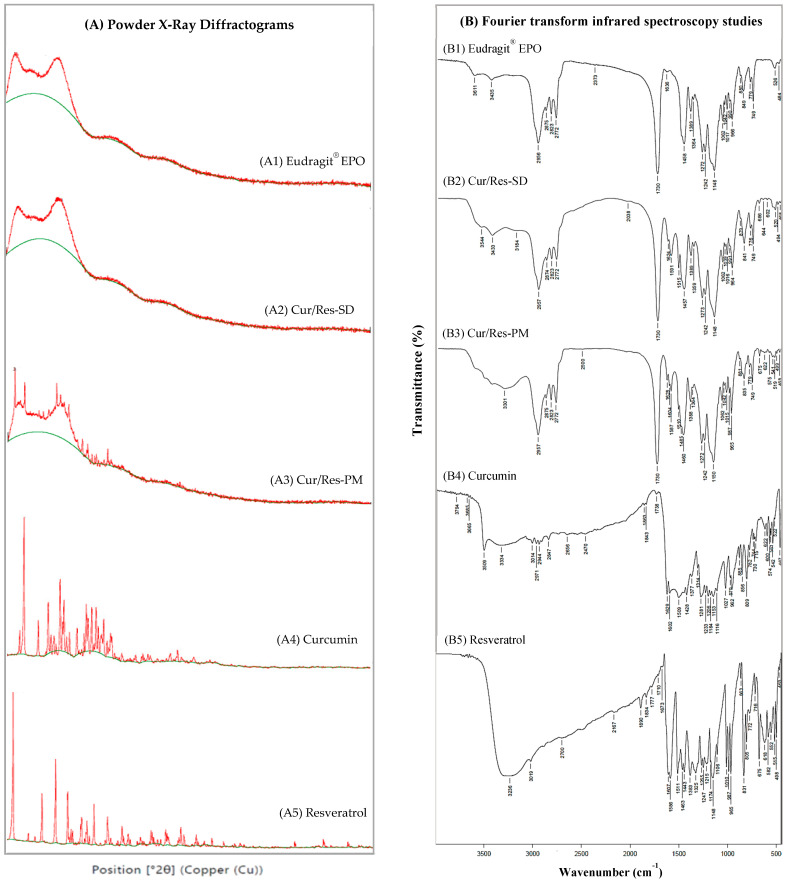
The powder X-ray diffractograms and Fourier transform infrared spectroscopy studies of curcumin, resveratrol, Eudragit^®^ EPO, Cur/Res-SD, and Cur/Res-PM. (**A**) Powder X-ray Diffractograms and (**B**) Fourier transform infrared spectroscopy study.

The powder X-ray diffraction patterns of curcumin, resveratrol, Eudragit^®^ EPO, Cur/Res-SD, and Cur/Res-PM are shown in Figure 2A. SDs and PMs were prepared using a Cur/Res: Eudragit EPO *w*/*w* ratio of 1:10. The important diffraction angles of curcumin occur at 7.90°, 8.94°, 12.21°, 14.53°, and 17.31°, whereas pure resveratrol showed peaks at 6.62°, 13.26°, 16.38°, 19.21°, 25.26°, and 28.32°, revealing the crystalline nature of both compounds. The hydrophilic polymer Eudragit^®^ EPO showed a halo pattern on a broad background, indicating the amorphous state of the polymer. Cur/Res-PM showed prominent peaks at positions (*2θ*) 6.63°, 8.91°, and 16.35°, and weaker signals at 17.36°, 19.21°, 22.41°, 23.66°, and 28.39° *2θ* values, indicating crystal presence. Peaks indicative of crystallinity were absent in diffractograms of Cur/Res-SD, indicating the transformation of curcumin and resveratrol from a crystalline to amorphous state, due to hydrogen bonding and strong intermolecular interactions between hydrophilic Eudragit^®^ EPO and both compounds. This behavior results in the enhancement of the solubility of curcumin and resveratrol, compared to physical mixtures and unformulated compounds. 

#### 3.1.3. Fourier Transform Infrared Spectroscopy Studies (FTIR)

FTIR analysis was performed to provide information on the physicochemical interactions between curcumin, resveratrol, and the hydrophilic carrier polymer, Eudragit^®^ EPO, in solid dispersions and physical mixtures (Figure 2B). SDs and PMs were prepared using a Cur/Res: Eudragit^®^ EPO *w*/*w* ratio of 1:10. The FTIR spectrum of curcumin featured a broad peak at 2470–3509 cm^−1^, indicating an O-H stretching vibration of hydroxyl groups in the polyphenolic compound. Peaks in the range of 1428–1602 cm^−1^ are assigned to the C=C stretching vibrations of alkene groups, while the peak at 1629 cm^−1^ denotes C=O stretching in ketone groups. Resveratrol as a polyphenolic compound gives rise to peaks at 3236 cm^−1^, 1443–1607 cm^−1^, and 965–1010 cm^−1^, due to O-H stretching vibrations, C=C stretching of phenyl groups, and C-O stretching, respectively.

The FTIR spectrum of Eudragit^®^ EPO exhibited a peak at 1730 cm^−1^, associated with C=O stretching of the ester carbonyl group. Other prominent peaks were detected at 1148 cm^−1^ (C-N stretching of dimethyl amino group) and 2956 cm^−1^ (N-H stretching of dimethyl amino groups). The FTIR spectra of Cur/Res-PM displayed a similar pattern to Eudragit^®^ EPO, indicating no significant interactions between the polyphenols and Eudragit.

The FTIR spectra of Cur/Res-SDs showed intermolecular interaction between the hydroxyl groups of the polyphenolic compounds (curcumin and resveratrol) and the cationic functional groups of Eudragit^®^ EPO that was revealed by the disappearance of the O-H stretching vibration at 2470–3509 cm^−1^ for curcumin and 965–1010 cm^−1^ for resveratrol, as well as the C=O stretching band at 1730 cm^−1^ for Eudragit^®^ EPO.

### 3.2. Physicochemical Characteristics of In Situ Gelling, Liquid Formulations Containing Cur/Res-SD

**Figure 3:**
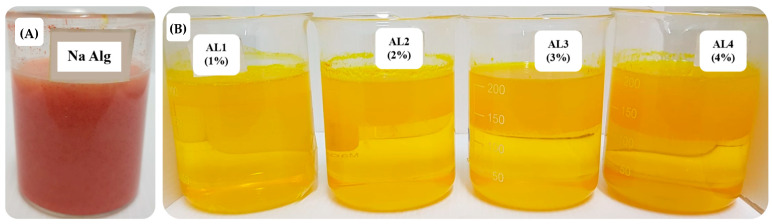
Physical appearance of in situ gelling, liquid formulations containing Cur/Res-SD. Gelling polymer: sodium alginate. (**A**) Fresh preparation and (**B**) after forming floating in situ gels in hydrochloric acid solution (0.1 N; pH = 1.2).

The physicochemical properties of in situ gelling, liquid formulations incorporating the curcumin–resveratrol solid dispersions are illustrated in Table 3. The in situ gelling, liquid formulations containing Cur/Res-SD presented as reddish-orange viscous liquids. (Figure 3) They were easily poured and rapidly transformed to floating raft structures on contact with SGF. The liquid, in situ gelling formulations changed to a yellow color, due to the keto-enol tautomerism dependent on the pH of the solution [45].

#### 3.2.1. pH of In Situ Gelling, Liquid Formulations Containing Cur/Res-SD

The pH of all in situ gelling, liquid formulations ranged between 8.7 and 9.5, the alkalinity being due to the presence of sodium bicarbonate and calcium carbonate in the formulations. According to the British Pharmacopoeia Volume III, alginate raft-forming oral suspensions should exhibit a pH between 7.0 and 9.5, as shown in Table 3. All in situ gelling formulations displayed acceptable pH values to maintain the formulation in liquid form and could be applied in further analysis.

#### 3.2.2. Floating Behavior of In Situ Gelling, Liquid Formulations Containing Cur/Res-SD

The addition of the liquid formulations to SGF resulted in rapid gel formation and flotation as a raft structure, due to the entrapment of CO_2_ in the gel matrix following reaction of CaCO_3_, and NaHCO_3_ with SGF (pH = 1.2). Furthermore, calcium ions released from calcium carbonate interacted with the anionic polysaccharide polymers (sodium alginate, pectin, and gellan gum) to crosslink and stabilize the hydrogel network.

The floating lag time (FLT) of the liquid formulations ranged from 3 to 173 s depending on the type and concentration of the gelling polymer used for preparation (Table 3). The floating lag time was increased relative to the increment of the gelling polymer concentration. The increased amount of gelling polymer hindered the dissolution of calcium carbonate (CaCO_3_) and interfered with the crosslinking of the divalent cations in anionic polymeric chains for gelation. Moreover, higher polymer concentrations result in the creation of a dense and cohesive polymeric structure. The entanglement network released carbon dioxide (CO_2_) at a slower rate.

The optimum floating lag time was set at less than 1 min, meaning that liquid formulations prepared using 1 and 2% *w*/*v* sodium alginate (medium and low viscosity), pectin (1 and 2%), and gellan gum met the criterion. Gellan gum, in particular, showed rapid floatation within a minute. All raft structures formed by in situ gelation of the liquid formulations remained afloat in SGF for more than 24 h, indicating efficient gastroretentive behavior for the delivery of curcumin and resveratrol.

#### 3.2.3. Density of Rafts Formed by In Situ Gelling, Liquid Formulations Containing Cur/Res-SD

The raft density must be less than the density of the gastric content (1.004–1.01 g/cm^3^) [41,42] to achieve flotation and to resist movement to the pyloric orifice and small intestine, thereby sustaining the local release of curcumin and resveratrol. The raft may also act as a physical barrier to prevent acid reflux into the esophagus. The raft density tended to increase with increasing concentration of the gelling polymers, but remained in the range of 0.24 to 0.62 g/cm^3^ (Table 3) due to the entrapment of CO_2_ in the gel matrix following reaction of CaCO_3_, and NaHCO_3_ with SGF.

#### 3.2.4. Raft Strengths Formed by In Situ Gelling, Liquid Formulations Containing Cur/Res-SD

Raft strength was affected by the type and concentration of gelling polymer in the liquid formulation (Table 3). The maximum raft strength was in the following order: gellan gum < low-viscosity sodium alginate < pectin < medium-viscosity sodium alginate. The highest raft strength (38.6 g) was recorded for formulations prepared using 4% *w*/*v* medium-viscosity sodium alginate, indicating effective crosslinking of the hydrogel network by Ca^2+^ ions. Similar results were reported previously for in situ gelling formulations based on alginate and incorporating curcumin [33], glycoside-rich *Centella asiatica* extract [38], and quercetin [37]. The lowest raft strength (0.4 g) was recorded for liquid formulations prepared using 1% low-viscosity sodium alginate. The raft strength of formulations based on pectin increased from 5.0 to 18.9 g on raising the concentration from 1 to 4%, due to increasing crosslink density in the formed hydrogel. Lower gellan gum concentrations (0.25% to 1% *w*/*v*) resulted in correspondingly lower raft strengths (3.2 to 5.0 g).

According to British Pharmacopoeia 2018, an acceptable raft strength is not less than 7.5 g, in order to resist breakdown by peristaltic movement in the gastrointestinal tract and to remain in the stomach. The floating in situ gelling formulations based on medium-viscosity sodium alginate exhibited a higher raft strength (10.1–38.6 g) than curcumin raft-forming formulations (4.1–6.0 g), as previously reported by Kerdsakundee et al. (2015) [33]. Thus, the liquid formulations containing medium-viscosity sodium alginate or pectin would be considered most suitable for producing gastroretentive, raft-forming delivery vehicles for curcumin and resveratrol.

#### 3.2.5. Viscosity

**Figure 4:**
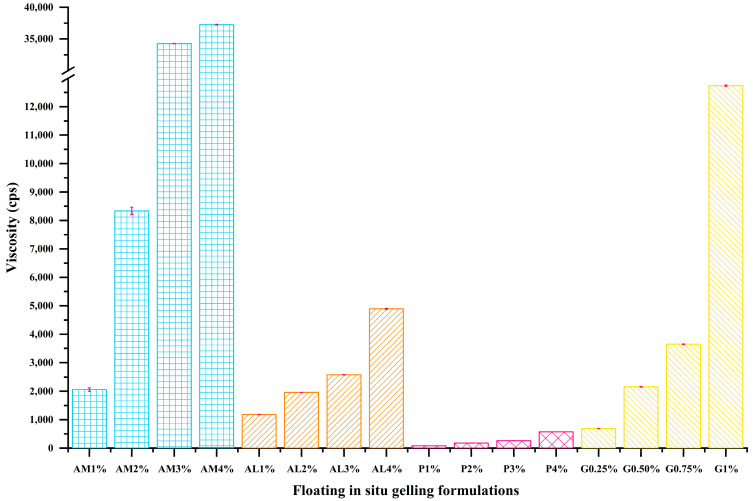
The viscosity measurement of floating in situ gel formulations.

**Figure 5:**
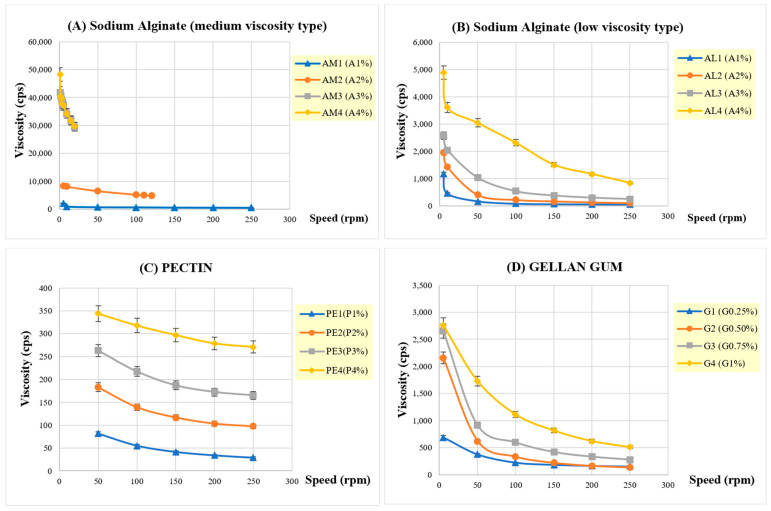
Rheological behavior of in situ gelling, liquid formulations containing Cur/Res-SD. (**A**) gelling polymer: sodium alginate, medium viscosity (**B**) gelling polymer: sodium alginate, low viscosity (**C**) gelling polymer: pectin (**D**) gelling polymer: gellan gum.

The viscosity of in situ gelling, liquid formulation systems is important for oral administration and should facilitate pouring. The viscosity of the formulations increased with increasing concentration of the gelling polymer (Figure 4). Low-viscosity sodium alginate, for example, resulted in a significant increase in viscosity from approximately 1000 to 5000 cps, on raising the concentration from 1 to 4%. Use of the medium-viscosity alginate in the formulation at 4% concentration produced a viscosity of 50,000 pcs, due to more extensive polymer chain entanglement, leading to a higher resistance to flow. The use of pectin as the gelling polymer at 1–4% *w*/*v* concentration resulted in liquid formulations with the lowest viscosity in the range of 82 to 577 cps. All in situ gelling, liquid formulations exhibited non-Newtonian, pseudoplastic flow or shear thinning behavior, where viscosity decreases with increasing shear rate (Figure 5).

#### 3.2.6. Release of Curcumin and Resveratrol from In Situ Gelling, Liquid Formulations Containing Cur/Res-SD

**Figure 6:**
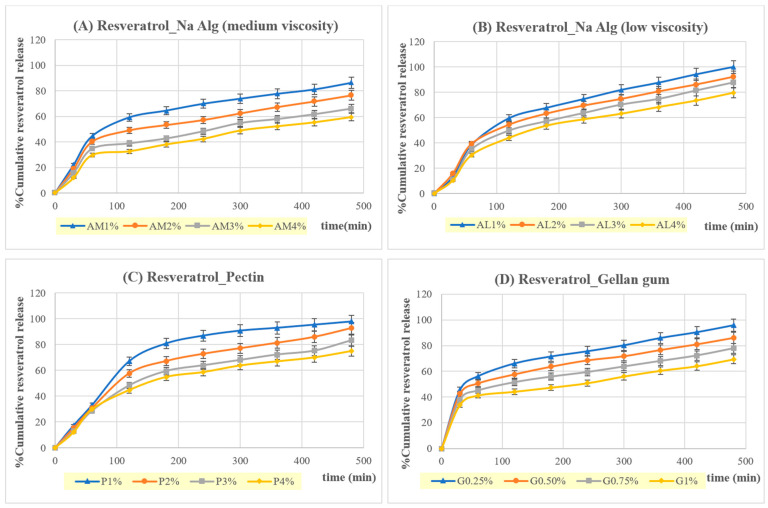
Cumulative resveratrol release from floating in situ gelling, liquid formulations containing Cur/Res-SD in SGF at 37 °C.

**Figure 7:**
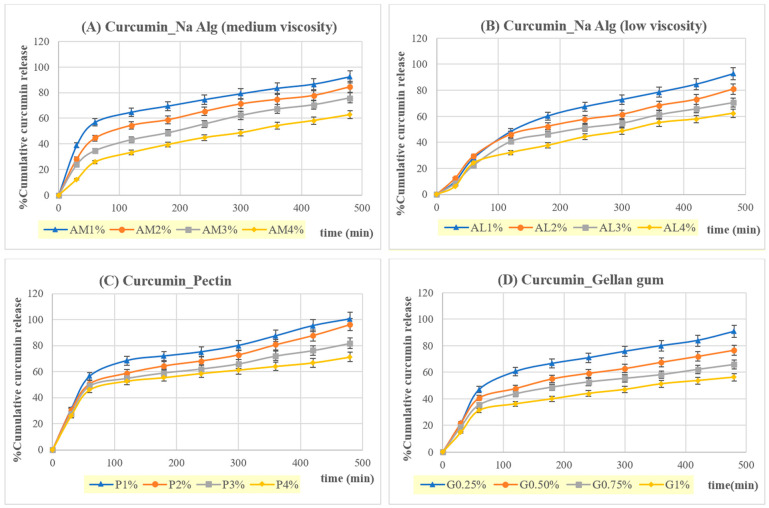
Cumulative curcumin release from in situ gelling, liquid formulations containing Cur/Res-SD in SGF at 37 °C.

The release profiles of curcumin and resveratrol for in situ gelling, liquid formulations incorporating Cur/Res-SD are shown in Figure 6 and Figure 7. SGF (pH = 1.2) comprised the release medium. The release behavior was affected by the type and concentration of gelling polymer. In general, all formulations investigated resulted in a gradual and highly efficient release of 60–100% of the curcumin and resveratrol content over 8 h and the release amount increased consistently as the concentration of gelling polymer decreased. 

Curcumin release was rapid over the first hour, for formulations based on pectin, gellan gum, and low-viscosity sodium alginate, followed by a phase of gradual release over the following 7 h. At least 50% of the curcumin load was released in 1 h from formulations based on these gelling polymers at 1% *w*/*v* concentration. However, formulations based on medium-viscosity sodium alginate tended to exhibit a gradual release of curcumin from the start of testing, indicating the presence of a lower-density hydrogel, which did not hinder curcumin transport.

The release behavior of resveratrol from the in situ gelling, liquid formulations was similar to that of curcumin. However, a gradual release from the start of testing combined with a highly efficient delivery was obtained for formulations prepared using medium-viscosity sodium alginate and pectin. Based on these findings, liquid formulations prepared from medium-viscosity sodium alginate (1% *w*/*v*, AM1) were selected for further study.

The kinetics of curcumin and resveratrol release were analyzed via linear regression, using the zero order, first order, Higuchi, and Korsmeyer–Peppas models. Resveratrol release from formulations based on pectin or sodium alginate (low viscosity) was best fitted to first-order kinetics. However, resveratrol release from gellan gum-based formulations tended to fit with the Korsmeyer–Peppas model. Curcumin release showed the best fit with the Higuchi model.

Liquid formulations based on 1% *w*/*v* medium-viscosity sodium alginate (AM1) provided the highest correlation with first-order kinetics and the Higuchi model for resveratrol, and with the Kormeyer–Peppas model for curcumin, suggesting a diffusion-controlled release mechanism. Curcumin and resveratrol release from raft-forming systems based on low-viscosity sodium alginate (AL1) had release exponents of 0.6473 and 0.7315 (0.45 < n ≤ 0.89) in the Korsmeyer–Peppas model and indicated a combination of diffusion and erosion mechanisms. The kinetic modeling of curcumin release from raft-forming formulations based on medium-viscosity sodium alginate and containing both curcumin and resveratrol is in agreement with a previous study, which incorporated curcumin–Eudragit^®^ EPO SDs alone in raft-forming formulations [33].

In situ gelling, liquid formulations containing medium-viscosity sodium alginate (1% *w*/*v*, AM1) were selected for an in vitro assay of cytotoxic and anti-inflammatory activity, due to a favorable combination of floating behavior, viscosity, and the sustained release of curcumin and resveratrol in SGF in excess of 80% over 8 h.

### 3.3. Cytotoxic Activity of In Situ Gelling, Liquid Formulations Containing Cur/Res-SD

Numerous studies have reported that curcumin can inhibit the invasion and proliferation of gastric cancer cells [46] through multiple signaling pathways, including the induction of apoptosis and a reduction in the chemo-resistance of cancer cells. Curcumin, a bioactive compound found in turmeric, has attracted considerable attention due to its potential health benefits, including anti-cancer properties. Resveratrol has also been reported to display anti-tumor activity against gastric cancer and to suppress the proliferation of tumor cells through the NF-κB signaling pathway [1,19]. The combination of curcumin and resveratrol has been reported to enhance the tumor suppressor activity of p53. Hence, dual loading in gastroretentive devices presents potential therapeutic advantages.

The cytotoxic activity of curcumin alone; resveratrol alone; curcumin/resveratrol mixture (CUR+RES); Cur/Res-SD; and in situ gelling, liquid formulation based on medium-viscosity sodium alginate and containing Cur/Res-SD (AM1) was evaluated using the AGS cell line. The liquid formulation based on medium-viscosity sodium alginate without curcumin and resveratrol served as the negative control.

Curcumin is observed to have anti-AGS activity, with an IC_50_ value of 7.10 ± 0.13 µg/mL, which is consistent with prior research, indicating its ability to impede cancer cell growth, induce apoptosis (programmed cell death), and hinder tumor progression.

Resveratrol has been investigated for its anti-cancer potential as well. With an IC_50_ value of 19.01 ± 0.37 µg/mL in this assay, its moderate anti-AGS activity suggests an inhibitory effect on AGS cells. Resveratrol’s interference with various signaling pathways involved in cancer development and progression may underlie its observed activity against AGS cells.

The mixture of curcumin and resveratrol displayed a higher cytotoxic activity against AGS cells (IC_50_ 6.08 ± 0.18 µg/mL) than the resveratrol substance (IC_50_ 19.01 ± 0.37 µg/mL) and Cur/Res-SD (IC_50_ 9.46 ± 0.13 µg/mL), as shown in Table 4. This synergistic effect implies that the combination may act through complementary mechanisms, enhancing their individual anti-AGS activities. The synergy between curcumin and resveratrol, often attributed to their modulation of multiple molecular targets in cancer biology, has been reported for the curcumin/resveratrol self-microemulsifying system, as reported by Jaisamut et al. (2017) [25]. Based on these findings, Cur/Res-SD-based floating in situ gelling formulations could help to decrease the required amount of each compound and enhance their therapeutic effectiveness.

However, the cytotoxic activity of curcumin and resveratrol formulated as in situ gelling, liquid formulations (AM1) exhibited a significantly greater cytotoxic activity against gastric adenocarcinoma cells (IC_50_ 0.46 ± 0.01 µg/mL) than the individual ‘free’ compounds, the Cur/Res mixture, or when curcumin and resveratrol were formulated as SDs with Eudragit^®^ EPO. This result indicates a robust inhibitory effect on AGS cells at a remarkably low concentration. Moreover, this behavior suggests that the gradual release of the polyphenols in amorphous form is responsible for the enhanced cytotoxicity. Further exploration of the composition and mechanism of action of AM1 could unveil novel therapeutic avenues for gastric cancer treatment.

In summary, the findings underscore the anti-AGS activity of curcumin, resveratrol, their combination (CUR+RES), and the AM1 compound, with varying degrees of potency. These results advocate for further investigation into natural compounds and combination therapies as promising strategies for managing gastric diseases.

### 3.4. Anti-Inflammatory Activity of In Situ Gelling, Liquid Formulations Containing Cur/Res-SD

The results of the anti-inflammatory assay are shown in Table 4. Curcumin exhibited potent anti-inflammatory activity (IC_50_ value of 3.08 ± 0.02 µg/mL), owing to its multiple mechanisms of action. It modulates various inflammatory pathways by inhibiting COX enzymes, reducing pro-inflammatory cytokines expression, and suppressing nuclear factor kappa B (NF-κB) activation. Curcumin’s ability to mitigate inflammation presents a compelling rationale for its potential anti-AGS activity.

Although displaying moderate anti-inflammatory activity (IC_50_ value of 22.92 ± 0.53 µg/mL), resveratrol exerts anti-inflammatory effects by inhibiting COX enzymes, modulating inflammatory cytokines, and suppressing NF-κB signaling. Formulations enhancing resveratrol’s solubility and bioavailability could potentiate its anti-AGS and anti-inflammatory effects, making it a promising candidate for gastric ulcer management.

The similar IC_50_ values for curcumin (3.08 ± 0.02 µg/mL), mixtures of curcumin and resveratrol (2.96 ± 0.03 µg/mL), and Cur/Res-SD (4.44 ± 0.03 µg/mL) suggested that the anti-inflammatory activity was due to curcumin. The combination of curcumin and resveratrol had a greater anti-inflammatory activity than resveratrol alone. Curcumin’s inhibition of COX enzymes and NF-κB signaling synergizes with resveratrol’s modulation of inflammatory cytokines, resulting in enhanced anti-inflammatory efficacy. This synergistic interaction is important for multi-targeted approaches in inflammation management, to suggest a potential dual impact on anti-AGS activity through inflammation suppression. The in situ gelling, liquid formulation without curcumin and resveratrol showed IC_50_ values of more than 200 µg/mL, indicating the absence of anti- inflammatory activity due to the delivery vehicle.

Importantly, the anti-inflammatory activity of in situ gelling, liquid formulations containing Cur/Res-SD (IC_50_ 0.61 ± 0.03 µg/mL) was found to be far in excess of indomethacin (IC_50_ 36.83 ± 1.99 µg/mL), a well-established NSAID, demonstrating a benchmark for comparison, but also underscoring the importance of targeting inflammation in disease management. The formulation AM1 had the anti-inflammatory potency to inhibit gastric ulceration and gastric cancer progression by targeting inflammation. As in the case of cytotoxic behavior, the gradual release of the polyphenols in amorphous form may be responsible for the enhanced anti-inflammatory effect.

Thereby, formulations optimizing AM1’s solubility and release could improve their bioavailability, facilitating its efficient delivery to gastric cancer cells. According to their multi-modal mechanisms of action and leveraging formulation strategies to enhance solubility and delivery, floating in situ gelling formulations containing Cur/Res-SD are compelling candidates for further investigation in gastric disease therapy.

## 4. Conclusions

In situ gelling, liquid formulations containing a mixture of curcumin and resveratrol in the form of solid dispersions with Eudragit^®^ EPO could simultaneously deliver curcumin and resveratrol on exposure to SGF. The raft structures formed by liquid formulations prepared using gelling polymers at medium concentration (1% *w*/*v*) exhibited rapid flotation within 40 s and gradual release of over 80% of the curcumin and resveratrol content in 8 h. The in situ gelling, liquid formulation based on medium- viscosity sodium alginate resulted in a significantly greater cytotoxic activity against gastric adenocarcinoma cell lines (AGS) than mixtures of free curcumin and resveratrol. The liquid formulations also exhibited higher anti-inflammatory activity against RAW 264.7 macrophage cells than indomethacin. These findings demonstrate the potential of in situ gelling, raft-forming, liquid formulations containing Cur/Res-SD as gastroretentive dosage forms for the treatment of gastric diseases.

## Figures and Tables

**Table 1:** Compositions of curcumin and resveratrol solid dispersions and physical mixtures.

Ratio of Cur/Res: EPO SD or Cur/Res: EPO PM	Weight of Active Compound (g)		Weight of Carrier Polymer (g)
	Curcumin	Resveratrol	Eudragit^®^ EPO
1:0.5	2.00	2.00	2.00
1:1	1.50	1.50	3.00
1:3	0.75	0.75	4.50
1:5	0.50	0.50	5.00
1:6	0.50	0.50	6.00
1:8	0.50	0.50	8.00
1:10	0.50	0.50	10.00

**Table 2:** Compositions of in situ gelling, raft-forming formulations containing a 1:1 mixture of curcumin and resveratrol.

Formulations	Weight of Each Component (g) in 100 mL			
	Gelling Polymer	Cur/Res-SD (1:10)	CaCO_3_	NaHCO_3_
Sodium Alginate (Medium Viscosity)				
AM1	1.00	2.50	0.75	0.50
AM2	0			
AM3	3.00			
AM4	4.00			
Sodium Alginate (Low Viscosity)				
AL1	1.00	2.50	0.75	0.50
AL2	2.00			
AL3	3.00			
AL4	4.00			
Pectin				
P1	1.00	2.50	0.75	0.50
P2	2.00			
P3	3.00			
P4	4.00			
Gellan gum				
G1	1.00	2.50	0.75	0.50
G2	2.00			
G3	3.00			
G4	4.00			

**Table 3:** Physicochemical properties of in situ gelling, liquid formulations containing Cur/Res-SD.

**Sodium Alginate (Medium Viscosity)**	**Na Alg_Med**	**pH**	FLT * (s)	**Raft Strength (g)**	**Density (g/mL)**
AM1	1.00%	9.34 ± 0.01	3.33 ± 0.47	10.10 ± 0.16	0.240 ± 0.004
AM2	2.00%	9.37 ± 0.01	64.33 ± 1.25	19.20 ± 0.28	0.283 ± 0.005
AM3	3.00%	9.42 ± 0.00	82.67 ± 2.36	25.00 ± 0.14	0.377 ± 0.008
AM4	4.00%	9.49 ± 0.00	173.00 ± 0.82	38.57 ± 0.66	0.575 ± 0.002
**Sodium alginate (Low viscosity)**	**Na Alg_Low**	**pH**	**FLT (s)**	**Raft strength (g)**	**Density (g/mL)**
AL1	1.00%	9.43 ± 0.01	17.67 ± 0.47	0.40 ± 0.02	0.318 ± 0.004
AL2	2.00%	9.45 ± 0.00	77.33 ± 0.47	0.90 ± 0.02	0.431 ± 0.008
AL3	3.00%	9.47 ± 0.01	87.00 ± 0.82	2.08 ± 0.02	0.436 ± 0.006
AL4	4.00%	9.49 ± 0.01	112.67 ± 0.47	5.80 ± 0.08	0.465 ± 0.007
**Pectin**	**Pectin**	**pH**	**FLT (s)**	**Raft strength (g)**	**Density (g/mL)**
P1	1.00%	8.67 ± 0.01	26.00 ± 2.94	5.07 ± 0.09	0.333 ± 0.006
P2	2.00%	8.83 ± 0.00	47.67 ± 0.47	7.87 ± 0.05	0.394 ± 0.001
P3	3.00%	8.94 ± 0.00	94.00 ± 2.45	10.73 ± 0.09	0.486 ± 0.006
P4	4.00%	9.01 ± 0.01	154.67 ± 3.30	18.93 ± 0.38	0.615 ± 0.004
**Gellan gum**	**Gellan gum**	**pH**	**FLT (s)**	**Raft strength (g)**	**Density (g/mL)**
G1	0.25%	9.24 ± 0.00	8.33 ± 0.47	3.17 ± 0.05	0.358 ± 0.000
G2	0.50%	9.23 ± 0.01	10.33 ± 0.47	3.77 ± 0.05	0.399 ± 0.003
G3	0.75%	9.26 ± 0.00	14.00 ± 0.82	4.37 ± 0.05	0.437 ± 0.003
G4	1.00%	9.29 ± 0.00	37.67 ± 2.62	5.03 ± 0.09	0.518 ± 0.007

* FLT = floating lag time.

**Table 4:** Anti-inflammatory and anti-AGS activity of in situ gelling liquid formulations containing Cur/Res-SD.

Samples	Equivalent to Active Compounds	
	Anti-Inflammatory	Anti-AGS
	IC_50_ (µg/mL)	IC_50_ (µg/mL)
Indomethacin	36.83 ± 1.99	-
Curcumin	3.08 ± 0.02	7.10 ± 0.13
Resveratrol	22.92 ± 0.53	19.01 ± 0.37
CUR+RES	2.96 ± 0.03	6.08 ± 0.18
Cur/Res- 1:10 SD	4.44 ± 0.03	9.46 ± 0.13
AM1	0.61 ± 0.03	0.46 ± 0.01
AM1 Blank	>200	>200

## Data Availability

Data and results were reported in this article.
